# Supplementary material for: NCAPG Regulates Myogenesis in Sheep, and SNPs Located in Its Putative Promoter Region Are Associated with Growth and Development Traits
Source: Animals (Basel). 2023 Oct 11;13(20):3173. doi: 10.3390/ani13203173 (PMC10603679; doi:10.3390/ani13203173)
Supplement: Supplementary file 1 [file animals-13-03173-s001.zip › animals-2636756-supplementary.pdf]

Table S1 Summarized information and functional prediction of detect five mutations.

SNP	Position	Ref > Alt	Mutation type (Novel or Existed)	Distance to TSS (bp)	Core similarity score	Matrix similarity score	Sequence	Consequence
SNP1	Chr6: 42090126	T > A	rs424493003	-1726	NA	NA	NA	NA
SNP2	Chr6: 42090844	C > T	rs159958117	-1008	NA	NA	NA	NA
SNP3	Chr6:42091061	T > C	rs423376306	-791	1	0.867	aacatgCACGTttgtaa	Score-Change
					1	0.866	aacacgCACGTttgtaa	Score-Change
					1	0.945	ctaaACAACacgca	Gain of TFBS
					1	1	CACGCa	Gain of TFBS
SNP4	Chr6:42091075	C > T	rs417096593	-777	1	0.898	gtttgtaAATCAggcct	Gain of TFBS
SNP5	Chr6:42091109	T > A	rs430255987	-743	1	1	aATTAA	Gain of TFBS

Table S2 Association analysis between SNPs in *NCAP*G promoter region and growth and development traits.

Age	Traits	SNP1			SNP2			SNP3			SNP4			SNP5		
		Genotype	Mean	Standard deviation	Genotype	Mean	Standard deviation	Genotype	Mean	Standard deviation	Genotype	Mean	Standard deviation	Genotype	Mean	Standard deviation
At birth	body weight	A/A	3.39	0.24	C/C	3.28	0.13	C/C	3.39	0.24	C/C	3.28	0.13	A/A	3.09	0.11
		T/A	3.09	0.10	C/T	3.09	0.10	C/T	3.09	0.10	C/T	3.09	0.10	A/T	3.23	0.11
		T/T	3.29	0.14	T/T	3.39	0.24	T/T	3.27	0.13	T/T	3.39	0.24	T/T	3.54	0.32
	body height	A/A	**36.76[a]**	2.05	C/C	**30.48[b]**	1.12	C/C	**36.76[a]**	2.05	C/C	**30.48[b]**	1.12	A/A	30.14	0.95
		T/A	**29.50[b]**	0.84	C/T	**29.52[b]**	0.85	C/T	**29.56[b]**	0.85	C/T	**29.52[b]**	0.85	A/T	30.68	0.94
		T/T	**30.56[b]**	1.15	T/T	**36.76[a]**	2.05	T/T	**30.43[b]**	1.13	T/T	**36.76[a]**	2.05	T/T	32.80	2.75
	body length	A/A	**29.73[a]**	1.75	**C/C**	**25.98[ab]**	0.96	C/C	**29.73[a]**	1.75	C/C	**25.98[b]**	0.96	A/A	25.28	0.81
		T/A	**24.92[b]**	0.72	**C/T**	**24.91[b]**	0.72	C/T	**24.92[b]**	0.72	C/T	**24.91[b]**	0.72	A/T	25.91	0.80
		T/T	**26.01[b]**	0.98	**T/T**	**29.73[a]**	1.75	T/T	**25.96[ab]**	0.96	T/T	**29.73[a]**	1.75	T/T	28.10	2.33
	Shin circumference	A/A	**5.46[a]**	0.32	**C/C**	**4.73[b]**	0.17	C/C	**5.46[a]**	0.32	C/C	**4.73[b]**	0.17	A/A	4.59	0.15
		T/A	**4.50[b]**	0.13	**C/T**	**4.51[b]**	0.13	C/T	**4.51[b]**	0.13	C/T	**4.51[b]**	0.13	A/T	4.71	0.14
		T/T	**4.74[b]**	0.18	**T/T**	**5.46[a]**	0.32	T/T	**4.72[b]**	0.17	T/T	**5.46[a]**	0.32	T/T	5.07	0.42
One-month-old	body weight	A/A	9.35	0.67	C/C	8.75	0.36	C/C	9.35	0.67	C/C	8.75	0.36	A/A	8.29	0.31
		T/A	8.34	0.27	C/T	8.33	0.28	C/T	8.33	0.28	C/T	8.33	0.28	A/T	8.73	0.30
		T/T	8.76	0.37	T/T	9.35	0.67	T/T	8.75	0.37	T/T	9.35	0.67	T/T	9.58	0.88
	body height	A/A	**43.94[a]**	2.49	C/C	**37.18[b]**	1.36	C/C	**43.94[a]**	2.49	C/C	**37.18[b]**	1.36	A/A	36.32	1.16
		T/A	**35.10[b]**	1.02	C/T	**35.09[b]**	1.03	C/T	**35.12[b]**	1.03	C/T	**35.09[b]**	1.03	A/T	36.59	1.14
		T/T	**37.26[b]**	1.40	T/T	**43.94[a]**	2.49	T/T	**37.14[b]**	1.37	T/T	**43.94[a]**	2.49	T/T	39.90	3.34
	body length	A/A	**41.99[a]**	2.42	C/C	**36.15[b]**	1.33	C/C	**41.99[a]**	2.42	C/C	**36.15[b]**	1.33	A/A	34.98	1.12
		T/A	**34.21[b]**	0.99	C/T	**34.22[b]**	1.00	C/T	**34.24[b]**	1.00	C/T	**34.22[b]**	1.00	A/T	35.89	1.11
		T/T	**36.25[b]**	1.36	T/T	**41.99[a]**	2.42	T/T	**36.11[b]**	1.33	T/T	**41.99[a]**	2.42	T/T	38.57	3.24
	Shin circumference	A/A	**6.26[a]**	0.36	C/C	**5.38[b]**	0.20	C/C	**6.26[a]**	0.36	C/C	**5.38[a]**	0.20	A/A	5.20	0.17
		T/A	**5.09[b]**	0.15	C/T	**5.09[b]**	0.15	C/T	**5.10[b]**	0.15	C/T	**5.09[b]**	0.15	A/T	5.36	0.16
		T/T	**5.41[b]**	0.20	T/T	**6.26[a]**	0.36	T/T	**5.38[b]**	0.20	T/T	**6.26[a]**	0.36	T/T	5.72	0.48
Two-month-old	body weight	A/A	**16.21[a]**	1.07	C/C	**13.32[b]**	0.59	C/C	**16.21[a]**	1.07	C/C	**13.32[b]**	0.59	A/A	13.48	0.50
		T/A	**12.97[b]**	0.44	C/T	**13.08[b]**	0.44	C/T	**13.09[b]**	0.44	C/T	**13.08[b]**	0.44	A/T	13.40	0.49
		T/T	**13.53[b]**	0.60	T/T	**16.21[a]**	1.07	T/T	**13.31[b]**	0.59	T/T	**16.21[a]**	1.07	T/T	13.90	1.43
	body height	A/A	**48.40[a]**	2.86	C/C	**38.81[b]**	1.56	C/C	**48.40[a]**	2.86	C/C	**38.81[b]**	1.56	A/A	39.39	1.33
		T/A	**37.96[b]**	1.17	C/T	**38.24[b]**	1.18	C/T	**38.27[b]**	1.18	C/T	**38.24[b]**	1.18	A/T	39.39	1.31
		T/T	**39.36[b]**	1.60	T/T	**48.40[a]**	2.86	T/T	**38.76[b]**	1.57	T/T	**48.40[a]**	2.86	T/T	40.07	3.84
	body length	A/A	**48.88[a]**	2.88	C/C	**39.62[b]**	1.58	C/C	**48.88[a]**	2.89	C/C	**39.62[b]**	1.58	A/A	39.91	1.34
		T/A	**38.49[b]**	1.18	C/T	**38.64[b]**	1.20	C/T	**38.67[b]**	1.19	C/T	**38.64[b]**	1.20	A/T	39.95	1.32
		T/T	**39.94[b]**	1.62	T/T	**48.88[a]**	2.89	T/T	**39.57[b]**	1.58	T/T	**48.88[a]**	2.89	T/T	40.63	3.87
	Shin circumference	A/A	**6.84[a]**	0.41	C/C	**5.55[b]**	0.22	C/C	**6.84[a]**	0.41	C/C	**5.55[b]**	0.22	A/A	5.56	0.19
		T/A	**5.43[b]**	0.17	C/T	**5.45[b]**	0.17	C/T	**5.46[b]**	0.17	C/T	**5.45[b]**	0.17	A/T	5.67	0.19
		T/T	**5.60[b]**	0.23	T/T	**6.84[a]**	0.41	T/T	**5.54[b]**	0.22	T/T	**6.84[a]**	0.41	T/T	5.73	0.54
Three-month-old	body weight	A/A	**23.93[a]**	1.62	C/C	**19.64[b]**	0.89	C/C	**23.93[a]**	1.62	C/C	**19.64[b]**	0.89	A/A	19.52	0.75
		T/A	**18.63[b]**	0.66	C/T	**18.76[b]**	0.67	C/T	**18.76[b]**	0.67	C/T	**18.76[b]**	0.67	A/T	19.45	0.74
		T/T	**19.92[b]**	0.91	T/T	**23.93[a]**	1.62	T/T	**19.64[b]**	0.89	T/T	**23.93[a]**	1.62	T/T	20.74	2.18
	body height	A/A	**54.45[a]**	3.38	C/C	**43.62[b]**	1.85	C/C	**54.45[a]**	3.38	C/C	**43.62[b]**	1.85	A/A	43.48	1.57
		T/A	**41.42[b]**	1.38	C/T	**41.56[b]**	1.40	C/T	**41.60[b]**	1.40	C/T	**41.56[b]**	1.40	A/T	43.20	1.55
		T/T	**43.99[b]**	1.89	T/T	**54.45[a]**	3.38	T/T	**43.56[b]**	1.86	T/T	**54.45[a]**	3.38	T/T	46.38	4.55

	body length	A/A	**56.20**^{a}	3.50	C/C	**45.19**^{b}	1.92	C/C	**56.20**^{a}	3.50	C/C	**45.19**^{b}	1.92	A/A	44.46	1.63
		T/A	**42.48**^{b}	1.43	C/T	**42.64**^{b}	1.45	C/T	**42.67**^{b}	1.45	C/T	**42.64**^{b}	1.45	A/T	44.92	1.61
		T/T	**45.62**^{b}	1.96	T/T	**56.20**^{a}	3.50	T/T	**45.15**^{b}	1.92	T/T	**56.20**^{a}	3.50	T/T	46.77	4.71
	Shin circumference	A/A	**7.22**^{a}	0.46	C/C	**6.03**^{b}	0.25	C/C	**7.22**^{a}	0.46	C/C	**6.03**^{b}	0.25	A/A	5.85	0.22
		T/A	**5.66**^{b}	0.19	C/T	**5.68**^{b}	0.19	C/T	**5.68**^{b}	0.19	C/T	**5.68**^{b}	0.19	A/T	5.97	0.21
		T/T	**6.07**^{b}	0.26	T/T	**7.22**^{a}	0.46	T/T	**6.02**^{b}	0.26	T/T	**7.22**^{a}	0.46	T/T	6.47	0.62
Six-month-old	body weight	A/A	**32.99**^{a}	2.37	C/C	**28.46**^{ab}	1.30	C/C	**32.99**^{a}	2.37	C/C	**28.46**^{b}	1.30	A/A	**29.47**^{a}	1.09
		T/A	**25.69**^{c}	0.97	C/T	**25.92**^{b}	0.98	C/T	**25.95**^{b}	0.98	C/T	**25.92**^{b}	0.98	A/T	**25.74**^{ab}	1.08
		T/T	**29.01**^{b}	1.33	T/T	**32.99**^{a}	2.37	T/T	**28.42**^{b}	1.30	T/T	**32.99**^{a}	2.37	T/T	**25.23**^{a}	3.16
	body height	A/A	**58.21a**	3.76	C/C	**47.33**^{b}	2.06	C/C	**58.21**^{a}	3.76	C/C	**47.33**^{b}	2.06	A/A	**50.16**^{a}	1.74
		T/A	**43.65**^{b}	1.54	C/T	**43.98**^{b}	1.56	C/T	**44.02**^{b}	1.56	C/T	**43.98**^{b}	1.56	A/T	**43.49**^{ab}	1.71
		T/T	**48.12**^{b}	2.11	T/T	**58.21**^{a}	3.76	T/T	**47.27**^{b}	2.07	T/T	**58.21**^{a}	3.76	T/T	**41.33**^{b}	5.02
	body length	A/A	**60.81**^{a}	3.98	C/C	**50.71**^{b}	2.18	C/C	**60.81**^{a}	3.98	C/C	**50.71**^{b}	2.18	A/A	**52.73**^{a}	1.84
		T/A	**46.03**^{b}	1.63	C/T	**46.39**^{b}	1.65	C/T	**46.44**^{b}	1.65	C/T	**46.39**^{b}	1.65	A/T	**46.39**^{ab}	1.81
		T/T	**51.58**^{c}	2.23	T/T	**60.81**^{a}	3.98	T/T	**50.64**^{b}	2.19	T/T	**60.81**^{a}	3.98	T/T	**44.30**^{a}	5.32
	Shin circumference	A/A	**7.26**^{a}	0.49	C/C	**6.24**^{b}	0.27	C/C	**7.26**^{a}	0.49	C/C	**6.24**^{ab}	0.27	A/A	6.35	0.22
		T/A	**5.52**^{b}	0.20	C/T	**5.56**^{b}	0.20	C/T	**5.57**^{c}	0.20	C/T	**5.56**^{b}	0.20	A/T	5.62	0.22
		T/T	**6.36**^{a}	0.27	T/T	**7.26**^{a}	0.49	T/T	**6.24**^{b}	0.27	T/T	**7.26**^{a}	0.49	T/T	5.53	0.65

Note: The shoulder labels a and b in the same column indicated that the difference was significant ($P < 0.05$), and no shoulder labels or the same shoulder label indicated no significant difference ($P > 0.05$).
